# Maximizing the performance of n-type Mg_3_Bi_2_ based materials for room-temperature power generation and thermoelectric cooling

**DOI:** 10.1038/s41467-022-28798-4

**Published:** 2022-03-02

**Authors:** Zihang Liu, Weihong Gao, Hironori Oshima, Kazuo Nagase, Chul-Ho Lee, Takao Mori

**Affiliations:** 1 International Center for Materials Nanoarchitectonics (WPI-MANA), National Institute for Materials Science (NIMS), Tsukuba, Japan; 2 National Institute of Advanced Industrial Science and Technology (AIST), Tsukuba, Ibaraki Japan; 3 Graduate School of Pure and Applied Sciences, University of Tsukuba, Tsukuba, Japan

**Keywords:** Thermoelectric devices and materials, Thermoelectrics

## Abstract

Although the thermoelectric effect was discovered around 200 years ago, the main application in practice is thermoelectric cooling using the traditional Bi_2_Te_3_. The related studies of new and efficient room-temperature thermoelectric materials and modules have, however, not come to fruition yet. In this work, the electronic properties of n-type Mg_3.2_Bi_1.5_Sb_0.5_ material are maximized via delicate microstructural design with the aim of eliminating the thermal grain boundary resistance, eventually leading to a high *zT* above 1 over a broad temperature range from 323 K to 423 K. Importantly, we further demonstrated a great breakthrough in the non-Bi_2_Te_3_ thermoelectric module, coupled with the high-performance p-type α-MgAgSb, for room-temperature power generation and thermoelectric cooling. A high conversion efficiency of ~2.8% at the temperature difference of 95 K and a maximum temperature difference of 56.5 K are experimentally achieved. If the interfacial contact resistance is further reduced, our non-Bi_2_Te_3_ module may rival the long-standing champion commercial Bi_2_Te_3_ system. Overall, this work represents a substantial step towards the real thermoelectric application using non-Bi_2_Te_3_ materials and devices.

## Abstract (web summary)

The awaited studies of new and efficient thermoelectric modules have not come to fruition yet. Here, the authors demonstrate a high thermoelectric performance of non-Bi_2_Te_3_ module for room-temperature power generation and thermoelectric cooling.

## Introduction

The long-term climate policy, an economy with net-zero greenhouse gas emissions by 2050, is significantly affecting the global economic situation, which stimulates the rapid development of clean and sustainable energy-related technologies^1^. Among them, thermoelectric effect can directly convert the temperature gradient to electricity and vice-versa. Despite the relatively low performance, thermoelectric devices have these unique advantages, including high reliability and scalability, no pollution and no noise, precise temperature control, enabling them suitable for a range of potential applications, such as aerospace power, refrigeration, and even energy harvesting^2–7^. The corresponding thermoelectric conversion efficiency or the cooling coefficient of performance (COP) is mainly determined by the dimensionless thermoelectric figure of merit (*zT*), which is defined as *zT* = [*S*^2^*σ*/(*κ*_*lat*_ + *κ*_*ele*_)]*T*, where *S*, *σ*, *κ*_*lat*_, *κ*_*ele*_, and *T* are Seebeck coefficient, electrical conductivity, lattice thermal conductivity, electronic thermal conductivity, and absolute temperature, respectively.

In general, the material’s thermoelectric performance is dominated by its crystal structure, specific composition, as well as nano-microstructure. Despite the intertwined relationship, the rational compromise and synergy among these abovementioned factors enable the realization of a maximum *zT* for a given material^8,9^. Previous investigations mainly focused on the modification of electronic band structure by elements alloying and/or tuning the phonon-scattering mechanism by designing special microstructure^10–16^. Very recently, the significance of grain-boundary resistance on the low-temperature charge transport has been recognized in some material systems^17–19^. This frequently overlooked effect leads to a lower charge carrier mobility and deteriorated practical *zT*s compared to their predicted values.

Bi_2_Te_3_ related alloys, which are discovered around the 1950 s as the best room-temperature thermoelectric material, are used in the commercial cooling area^20,21^. However, one of the main reasons that limit its further wide applications is the scarcity of Tellurium (Te) element. During the past decade, some new Te-free thermoelectric candidates with high *zT*s, such as α-MgAgSb^22–24^, Mg_3_Bi_2_^25–30^, SnSe^31,32^, enable the potential replacement of Bi_2_Te_3_, in which a high power-generation performance of Mg_3_Sb_2_/MgAgSb module, with the target for harvesting low-temperature waste heat, has already been demonstrated very recently^33,34^. P-type α-MgAgSb shows the large enhancement of thermoelectric performance at the low-temperature range by suitable doping^22,35^. Alloying with Mg_3_Bi_2_ in n-type Mg_3_Sb_2_ yields the optimum bandgap and simultaneously strengthens the point-defect scattering^25–28,36–45^, therefore contributing to highly competitive *zT*s. Moreover, a high-performance uni-couple (with p-type Bi_0.5_Sb_1.5_Te_3_) for thermoelectric cooling has been experimentally realized^25^. However, there is still no report about non-Bi_2_Te_3_ Peltier module thus far, due to a combination of the complicated synthesis method and the challenging module-fabrication process for these non-Bi_2_Te_3_ thermoelectric systems. Very recently, thermoelectric module consisting of n-type Bi_2_Te_3_ and p-type SnSe showed a maximum temperature difference *ΔT*_*max*_ of 45.7 K^32^, being attractive for future applications.

**Fig. 1:**
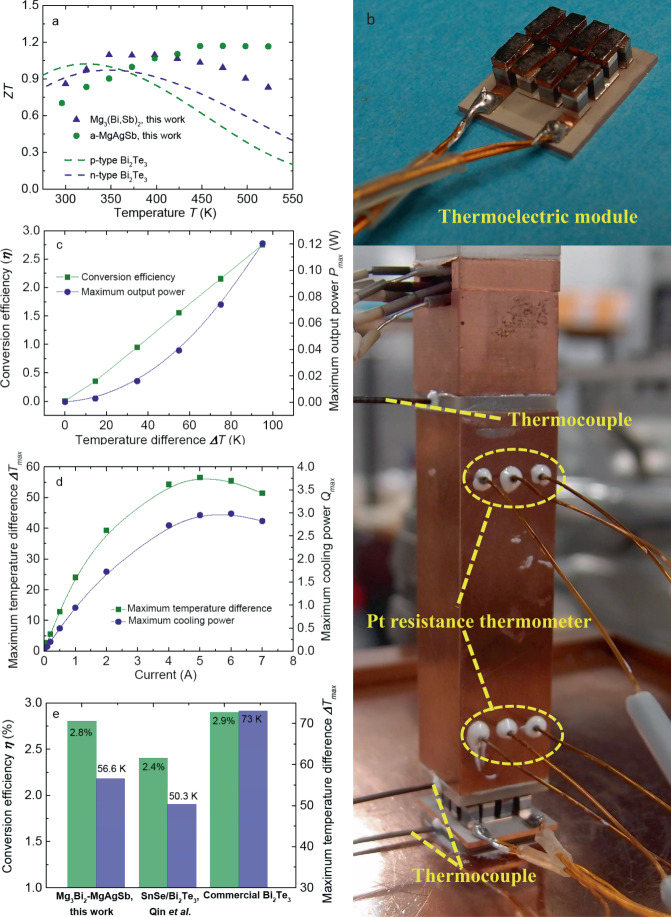
High-performance thermoelectric module, consisting of n-type Mg_3.2_Bi_1.5_Sb_0.498_Te_0.002_Cu_0.01_ and p-type α-Mg_0.99_Cu_0.01_Ag_0.97_Sb_0.99_, for room-temperature power generation and cooling application. **a** Temperature-dependent *zT* of both n-type and p-type materials in comparison to commercial Bi_2_Te_3_ data^8^, (**b**) images of both the fabricated module and the cooling measurement setup, (**c**) the measured conversion efficiency *η* and the maximum output power *P*_*max*_ as a function of temperature difference *ΔT* with the cold-side temperature *T*_*c*_ as 278 K, (**d**) the measured maximum temperature difference *ΔT*_*max*_ and the maximum cooling power *Q*_*max*_ as a function of electric current *I* with the *T*_*h*_ as 323 K, (**e**) comparison of the conversion efficiency *η* (at the *ΔT* of about 95 K) and the measured maximum temperature difference *ΔT*_*max*_ (at the *T*_*h*_ of 323 K) among our fabricated module, SnSe module^32^, and commercial Bi_2_Te_3_ module (Zone-melting Bi_2_Te_3_ data from Deng et al.^46^ and high-performance RC3-8 module type from Marlow Industries, Inc.^47^, respectively. Here it should be noted that since the cooling-performance measurement of SnSe/Bi_2_Te_3_ module was performed at the *T*_*h*_ of 300 K, we used the normalized method to calculate the *ΔT*_*max*_ at the *T*_*h*_ of 323 K (*ΔT*_*max-323* *K*_ = 1.1 × *ΔT*_*max-300* *K*_) for a reasonable comparison, where the multiplying factor 1.1 is obtained based on the data collection from Marlow Industries, Inc.

Herein, the thermoelectric performance of Mg_3_(Bi, Sb)_2_ system was fully maximized through rational microstructural design to eliminate the intrinsic grain-boundary resistance, which contributed to a higher *zT* than the commercial n-type Bi_2_Te_3_ above room temperature (Fig. 1a). Coupled with the optimized p-type α-MgAgSb (Fig. 1a), we demonstrated a remarkably high thermoelectric performance of non-Bi_2_Te_3_ and 8-pairs module for room-temperature power generation and thermoelectric cooling. These images of both the fabricated module and the cooling-performance measurement setup were given here (Fig. 1b). Specifically, at the *ΔT* of 35 K, the *η* and the maximum output power *P*_*max*_ reached 0.95% and 0.016 W (Fig. 1c), respectively. Moreover, the peak *η* reached 2.8% associated with a *P*_*max*_ of 0.12 W at the *ΔT* of 95 K. The calculated output power density of our fabricated module, with a whole cross-sectional dimension of 18 mm × 15 mm, was comparable to the zone-melting Bi_2_Te_3_ module (Fig. S1)^46^. Meanwhile, for cooling applications, when working at the optimum electric current *I* with a hot-side temperature *T*_*h*_ of 323 K, the *ΔT*_*max*_ and the maximum cooling power *P*_*max*_ could reach 56.5 K and 3.0 W (Fig. 1d), respectively. The achieved thermoelectric performance of our fabricated module is higher than the recently reported high-performance SnSe/Bi_2_Te_3_ module^32^, even comparable to the commercial Bi_2_Te_3_^46,47^ (Fig. 1e). Considering the billion-US-dollar market for the global thermoelectric Peltier module, this work brings promise for non-Bi_2_Te_3_ thermoelectric cooling and therefore paves up the avenue for emerging cooling technology.

## Results and discussion

**Fig. 2:**
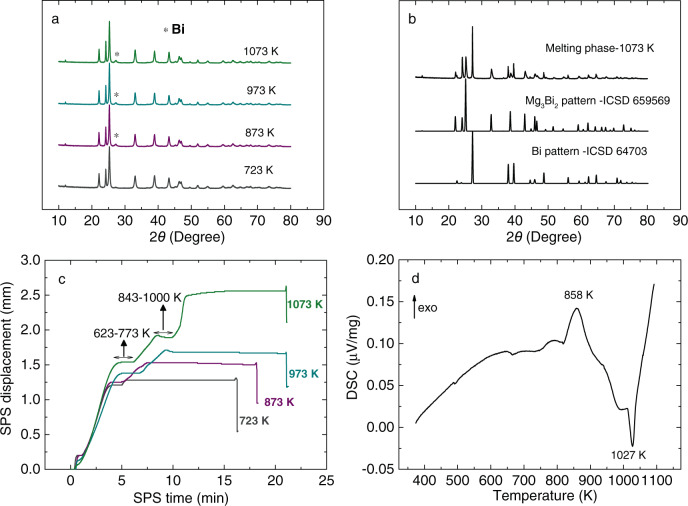
Phase characterization of Mg_3.2_Bi_1.5_Sb_0.498_Te_0.002_Cu_0.01_ samples at different sintering temperatures (723, 873, 973, and 1073 K). **a** XRD patterns, (**b**) melting-phase XRD pattern of the sample sintered at 1073 K. Here since the XRD pattern position of one melting phase is between Mg_3_Bi_2_ and Mg_3_Sb_2_, the Mg_3_Bi_2_ pattern is displayed for a simple comparison. **c** Spark plasma sintering (SPS) displacement as a function of sintering time, (**d**) Differential scanning calorimeter (DSC) measurement result of the sample sintered at 723 K.

To possess the optimum bandgap for the room-temperature application, it is imperative to shift the target material of Mg_3_(Bi, Sb)_2_ system from Sb rich to Bi rich. Hence, Mg_3_Bi_1.5_Sb_0.5_ is chosen as the basic composition, and doping with a little Te enables to tune the carrier concentration. Since minor Cu addition in Mg_3_Sb_1.5_Bi_0.5_ realized the remarkable enhancement of low-temperature *zT*^33^, herein the nominal composition is Mg_3.2_Bi_1.5_Sb_0.48_Te_0.002_Cu_0.01_. We revealed that the spark plasma sintering (SPS) temperature has a noticeable impact on the thermoelectric properties, which is beyond the conventional understanding that sintering temperature only affects the densification mechanism or main-phase change^48,49^. The phase feature when sintered at the different temperatures was thoroughly investigated by a combination of X-ray diffraction (XRD) analysis, in situ SPS displacement record, and differential scanning calorimetry (DSC) measurement (Fig. 2). Apparently, no impurity phase can be observed for the sample sintered at 723 K (Fig. 2a). In contrast, Bi impurity phase starts to appear at a higher sintering temperature than 723 K (Fig. S2). Meanwhile, the significant evaporation and the appearance of the squeezed melting phase are observed simultaneously (Fig. S3). Furthermore, XRD pattern of the melting-phase reveals that it consists of two different phases (Fig. 2b), namely Bi and Mg_3_(Bi, Sb)_2_. This should be related to the high-temperature decomposition of Mg_3_(Bi, Sb)_2_ due to its intrinsic instability, which is revealed through synchrotron powder XRD measurements along with synchrotron X-ray total scattering during repeated thermal cycling of Mg_3_Sb_1.475_Bi_0.475_Te_0.05_ powder reported by Jørgensen et al.^50^ This phenomenon can be further confirmed by the in situ recorded SPS displacement during the sintering process (Fig. 2c). For the sample sintered at 1073 K, there exist three independent plateaus that are distinct from other samples: the first one appears at the sintering-temperature range of 623–773 K, the second one during 843–1000 K, as well as the third one at the 1073 K isothermal process. These two melting phases are squeezed out during these abovementioned temperature ranges meanwhile Mg evaporation leads to the formation of nano-micro pores. As the utilization rate of raw materials is not 100%, the squeezed melting phase is associated with a little economic loss. For the heating DSC measurement (Fig. 2d), the exothermic peak at 858 K may be due to relaxing the lattice strain since the previous report claimed the origin of the crystalline Bi phase is caused by a reduction of microscopic strain in the structure^50^, while the subsequent endothermic reaction should be related to the severe partial decomposition reaction.

**Fig. 3:**
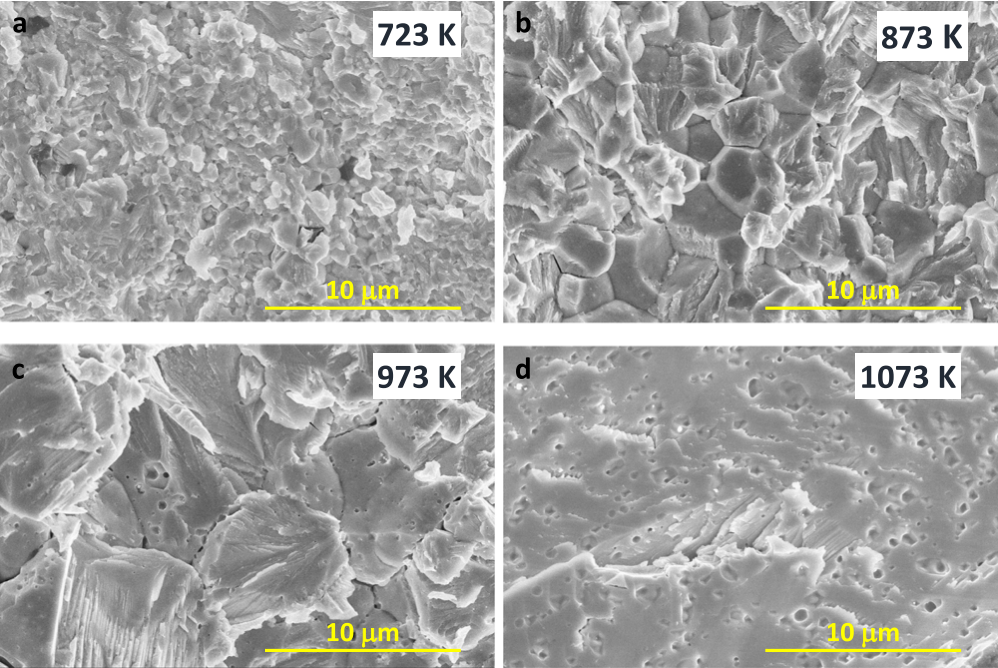
Microstructural evolution of Mg_3.2_Bi_1.5_Sb_0.498_Te_0.002_Cu_0.01_ samples at different sintering temperatures. **a** 723 K, (**b**) 873 K, (**c**) 973 K, and (**d**) 1073 K.

In addition to the phase component, tuning the sintering-temperature affects the microstructural feature considerably. As revealed by Scanning Electron Microscopy (SEM) analysis, the grain size has remarkably increased from several hundred nanometers (sintered at 723 K) to dozens of micrometers (sintered at 1073 K). In addition, the grain morphology has changed from the equiaxed structure (at 723, 873, and 973 K) to the columnar structure (at 1073 K) (Fig. 3), in which the columnar-structure characteristic can be observed in the corresponding low-magnification SEM image (Fig. S4). This is due to the liquid compact sintering effect at the high-temperature that is favorable to the formation of the large and anisotropic grain, as revealed in the previous report^51^. Simultaneously, sintering at 1073 K leads to the larger pores size and higher porosity as a direct consequence of Mg evaporation, whereas sintering at 723 K is unable to realize the dense compact, which can be reflected by the measured sample density (Table S1). Since Mg has a higher saturated vapor pressure than Sb and Bi at a defined temperature, the real chemical composition measured by SEM + EDS proved the Mg loss for sintering at high temperatures (Table S2), consistent with the annealing result of Mg_3_(Bi, Sb)_2_^52^. Clearly, the pores inside the sample start to appear when the sintering temperature is above 873 K (Fig. S5). In addition, the number of pores, namely the amount of evaporation, is gradually increased with the sintering temperature. However, it is difficult to observe the SPS displacement caused by the Mg evaporation during the sintering process since the impact of Mg evaporation is less significant compared to the melting-phase sequent out process.

**Fig. 4:**
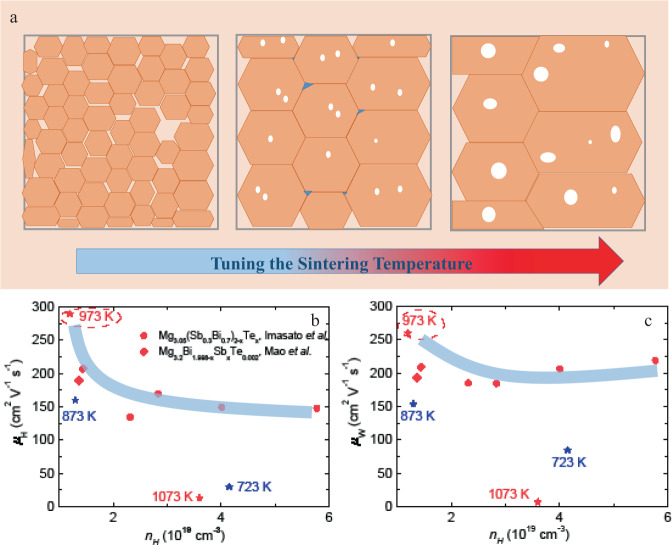
The effect of tuning the sintering temperature on the electronic properties of Mg_3.2_Bi_1.5_Sb_0.498_Te_0.002_Cu_0.01_ samples. **a** The schematic diagram of microstructural evolution when tuning the sintering temperature, (**b**, **c**) the measured charge carrier mobility *μ*_*H*_ values and the predicted weighted mobility *μ*_*W*_ as a function of carrier concentration *n*_*H*_, respectively, in comparison with the data from Mao et al. and Imasato et al.^25,26^.

The microstructural evolution originated from tuning the sintering-temperature effect was illustrated schematically (Fig. 4a). Briefly, sintering at the appropriate temperature realizes the large grain size with small pores and simultaneously minor Cu addition beneficially modifies the grain-boundary complexions with high electrical conductivity^33^, both of which contribute to the observed high charge carrier mobility *μ*_*H*_. A high *μ*_*H*_ around 290 cm^2^ V^−1^ s^−1^ can be realized in our work through rationally designing the microstructure (Fig. 4b), which is much higher than these reported Mg_3_Bi_2_ based samples at the same carrier concentration *n*_*H*_ from Mao et al.^25^ and Imasato et al.^26^ It should be noted that at the too-high sintering temperature (~at 1073 K), due to both the large pore size and the high porosity, *μ*_*H*_ deteriorates remarkably in turn that was about 22 times less compared to the maximum value. The weighted mobility *μ*_*W*_ is a good tool to assess the potential of the material’s electronic properties in thermoelectric^19,53^, which is independent of the doping element and/or real *n*_*H*_. Generally, *μ*_*W*_ is codetermined by the drift mobility *μ*_*D*_ and the density of states effective mass m^*^, shown in the following equation:

1 $${\mu }_{w}\,=\,{\mu }_{D}{\left(\frac{{m}^{\ast }}{{m}_{e}}\right)}^{3/2}$$

where *m*_*e*_ is the electron mass. The calculation method was according to the simplified mathematic model proposed by Zhu et al.^54^ (Table S3). Due to the high *μ*_*H*_, the sample (sintering at 973 K) possesses the highest *μ*_*W*_ in the Mg_3_(Bi, Sb)_2_ system that shares the same tendency with *μ*_*H*_ (Fig. 4c), indicative of the good electronic quality of our synthesized samples.

**Fig. 5:**
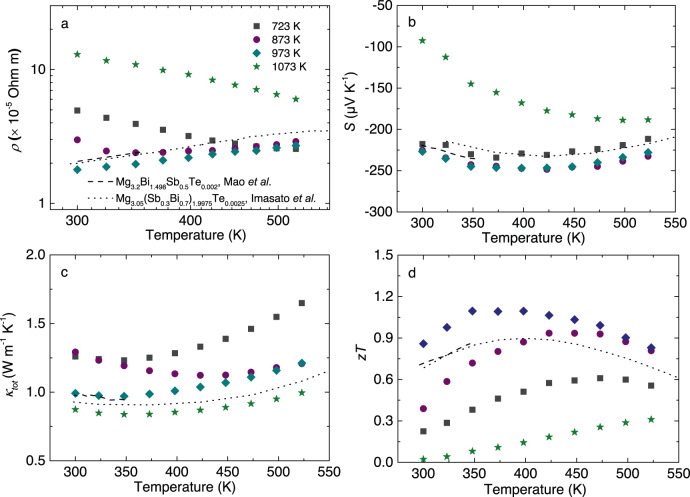
Temperature-dependent thermoelectric properties of Mg_3.2_Bi_1.5_Sb_0.498_Te_0.002_Cu_0.01_ samples at different sintering temperatures (723, 873, 973, and 1073 K), in comparison to the data from Mao et al. and Imasato et al.^25,26^. **a**–**d** Electrical resistivity *ρ*, Seebeck coefficient *S*, total thermal conductivity *κ*_*tot*_, and thermoelectric figure of merit *zT*, respectively.

Tuning the sintering temperature eliminates the thermal grain-boundary resistance and therefore observably reduces the electrical resistivity *ρ* at the low-temperature range (Fig. 5a). In comparison to these optimized samples from Mao et al. and Imasato et al.^25,26^ our synthesized sample (~973 K) shows the lowest *ρ* at the whole measured temperature range, due to the combined advantage from tuning the sintering temperature and minor Cu addition. However, the tendency of *n*_*H*_ as a function of sintering temperature is not regular, probably caused by the real-composition change and/or the severe decomposition process at 1073 K. Seebeck coefficient *S*, however, shows the anomalous tendency (Fig. 5b), irrespective of the *ρ* or *n*_*H*_. This is in good agreement with these previous reports in Mg_3_Sb_1.5_Bi_0.5_^55,56^. As a result of the reduced *ρ*, power factor *PF* shows a significant enhancement by optimizing the sintering temperature, in which the highest *PF* in our work surpasses 30 μW cm^−1^ K^−2^ (Fig. S6). Here it should be noted that the obtained *S* measurement results show some discrepancy between the commercial ZEM-3 and the homemade apparatus in the work of Imasato et al.^26^ probably due to the cold-finger effect, which may underestimate somewhat the measured *PF* from Imasato et al.^26^ Combined with the thermal conductivity *κ*_*tot*_ (Fig. 5c), tuning the sintering temperature significantly enhances the thermoelectric figure of merit *zT*, in which *zT* above 1 over a broad temperature range from 323 to 423 K is achieved accompanied with a high room-temperature *zT* close to 0.9 that is the record-high value in the Mg_3_Sb_2_ system (Fig. 5d).

**Fig. 6:**
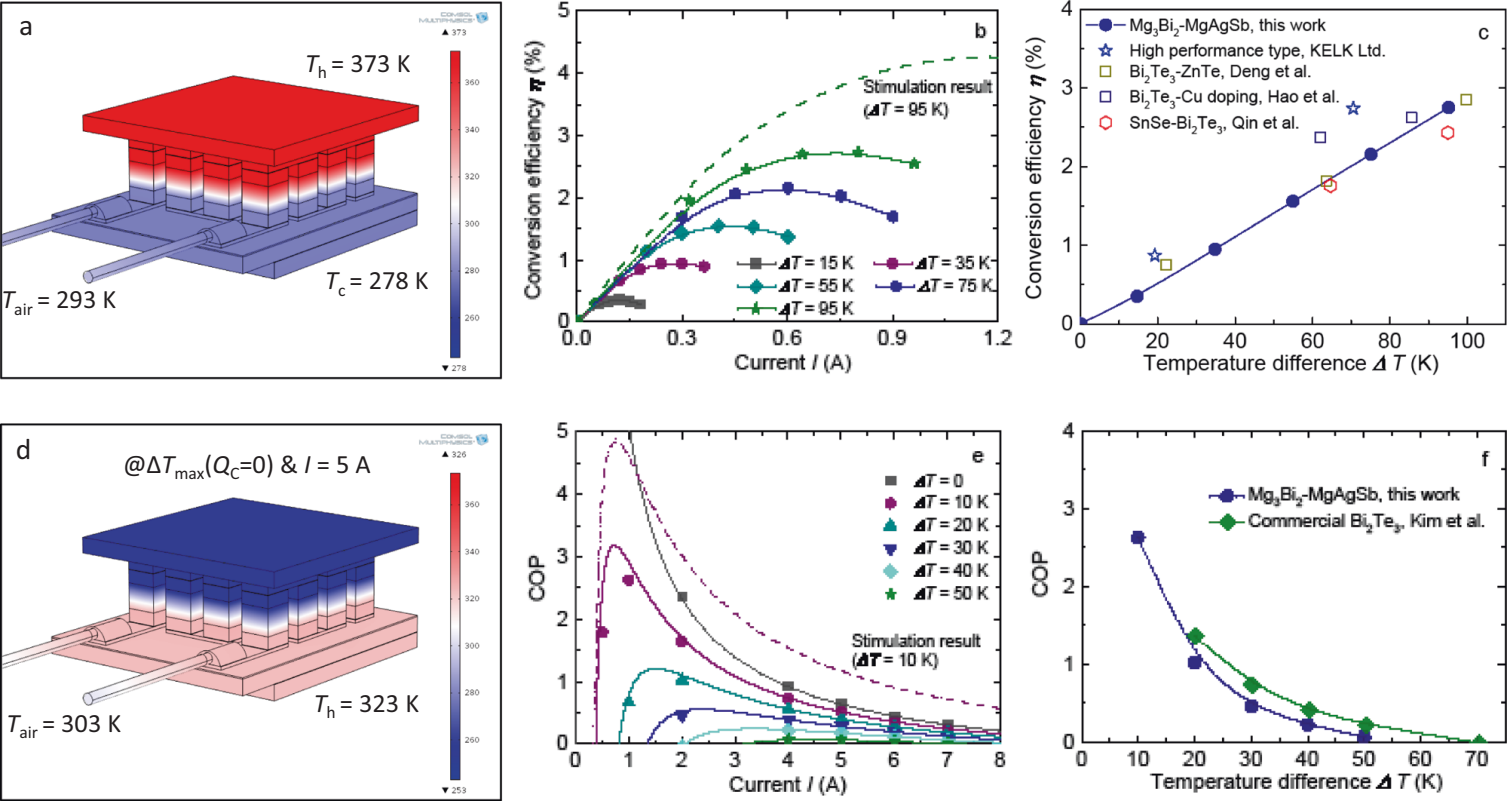
Thermoelectric performance of our fabricated Mg_3_Bi_2_–MgAgSb module. **a** Stimulation result of temperature distribution in the fabricated module for power generation using COMSOL Multiphysics^®^ software, (**b**) the measured conversion efficiency *η* as a function of electric current *I* at the temperature difference *ΔT* of 15, 35, 55, 75, and 95 K, (**c**) the measured *η* as a function of temperature difference  *ΔT*, compared to advanced Bi_2_Te_3_ modules from research lab^46,57^ and commercial Bi_2_Te_3_ module from KELK Ltd.^58^, as well as SnSe-Bi_2_Te_3_ module^32^, (**d**) stimulation result of the maximum temperature difference *ΔT*_*max*_ in the fabricated module for cooling using COMSOL Multiphysics® software, (**e**) the measured and the predicted COP as a function of electric current *I* at the temperature difference *ΔT* of 0, 10, 20, 30, 40, and 50 K, (**f**) comparison of the maximum COP between our fabricated module and commercial Bi_2_Te_3_ as a function of *ΔT*.

To further evaluate the potential of our synthesized material for the room-temperature thermoelectric application, an 8-pairs thermoelectric module, consisting of n-type Mg_3.2_Bi_1.5_Sb_0.498_Te_0.002_Cu_0.01_ (sintered at 973 K) and p-type α-Mg_0.99_Cu_0.01_Ag_0.97_Sb_0.99_, was designed and successfully fabricated. α-MgAgSb, as a high-performance and mechanically robust thermoelectric material, is a promising substitute candidate for p-type (Bi, Sb)_2_Te_3_^24^. Cu doping can further optimize thermoelectric properties, leading to a higher average *zT* (Fig. S7). Concerning the module details, the fabrication method and the corresponding measurement principle, as well as the stimulation theory of theoretical performance were given in the following methods part. First, we performed the three-dimensional finite-element simulations of both power-generation and cooling performance with COMSOL Multiphysics^®^ software in the Heat Transfer Module to optimize the leg geometry and length, shown in Figs. S8, S9, respectively. The theoretical temperature distribution of our fabricated module is obtained at the hot-side temperature of 373 K (Fig. 6a). Under the optimized *I* of 0.8 A, the maximum *η* is 2.8% at the hot-side temperature of 373 K, namely under the temperature difference of 95 K (Fig. 6b). This achieved high *η* can be comparable to the best Bi_2_Te_3_ module produced in a research laboratory^46,57^, but a little lower than the high-performance type commercial module from KELK Ltd^58^ (Fig. 6c). However, the difference between the measured value and theoretical stimulation (dash line in Fig. 6b) is still noticeable. This is primarily due to the larger internal resistance *R* as a consequence of the relatively large interfacial resistance between the interfacial layer and element material for both n-type and p-type legs, leading to the lower output power *P* and *η* in comparison to the theoretical stimulation (Fig. S10). Herein, the interfacial layers of n-type and p-type leg are pure Fe and Ag layers, respectively, both of which do not show the obvious voltage jump around the interface (Fig. S11). Besides, their corresponding EDS mapping images further indicate no observable elemental diffusion (Figs. S12, S13). The interfacial resistance of the present n-type and p-type legs is around 20 μΩ cm^−2^, a little higher than the threshold value of 5 μΩ cm^−2^ accepted for thermoelectric modules with a short leg around 1–2 mm^59^. The resistivity of n-type leg is approximately consistent with that of sample without interfacial layer. However, it is twice larger in p-type leg. The SPS condition must be optimized with elements adding interfacial layer for further improvement. In contrast, the open-circuit voltage *V*_*oc*_ and the maximum heat dissipated from the cold side of the module *Q*_*max*_ are basically consistent with the simulation results (Fig. S14), which indicates the good accuracy of *S* and *κ* measurement, respectively. Regarding thermoelectric cooling, the theoretical cooling temperature difference of our fabricated module is obtained at the condition of the hot-side temperature of 323 K, air temperature of 303 K, as well as the working current *I* of 5 A (Fig. 6d). Specifically, one of the most important indexes is the cooling coefficient of performance (COP) that represents the ratio of the amount of heat absorbed *Q*_*C*_ to the total electrical power input *P*, as COP = *Q*_*C*_/*P*. The maximum COP of our fabricated module, under the temperature difference of 10 and 20 K, reaches 2.6 and 1.0 (Fig. 6e), respectively, with the hot-side temperature *T*_*h*_ as 323 K. Importantly, an expected higher COP value, as well as a larger both *ΔT*_*max*_ and *Q*_*max*_ can be possibly achieved if the contact resistance can be further reduced (Figs. 6e, S15). Our achieved performance can be comparable with the commercial Bi_2_Te_3_ thermoelectric module (Fig. 6f). The demonstration of remarkably high thermoelectric performance in a non-Bi_2_Te_3_ module holds great promise for room-temperature power generation and emerging cooling applications.

In summary, we demonstrated the remarkably high thermoelectric performance of non-Bi_2_Te_3_ module, consisting of n-type Mg_3_Bi_1.5_Sb_0.5_ and p-type α-MgAgSb, for room-temperature power generation and thermoelectric cooling. Herein, the n-type material’s thermoelectric performance was maximized through rationally designing the microstructure by tuning the sintering temperature and minor Cu addition. The thermal grain-boundary resistance at the low-temperature range was completely eliminated that contributed to the record-high charge carrier mobility and thus significantly enhanced the electronic properties. Therefore, the achieved room-temperature *zT* was boosted to 0.9, in addition to a broad temperature plateau above 1 from 323 to 423 K. Our work highlights the importance of microstructure on the charge carrier transport and, more importantly, offers great hope for room-temperature thermoelectric application using non-Bi_2_Te_3_ materials and devices.

## Methods

### Materials synthesis

High-purity raw materials from Sigma-Aldrich company were directly weighed according to the nominal composition Mg_3.2_Bi_1.5_Sb_0.498_Te_0.002_Cu_0.01_, as well as Mg_1−x_Cu_x_Ag_0.97_Sb_0.99_ (*x* = 0 and 0.01), loaded into the ball-milling jaw in the glovebox, and finally subjected to milling process (SPEX SamplePrep 8000 Mixer Mill). The ball-milled powder was directly subjected to sintering without a pre-calcination process. For Mg_3.2_Bi_1.5_Sb_0.498_Te_0.002_Cu_0.01_, the ball-milling time is 5 h and the obtained nanopowders were loaded into the graphite die and sintered by spark plasma sintering (SPS, SPS-1080 System, SPS SYNTEX INC) at different sintering temperatures (723, 873, 973, and 1073 K) with the pressure of ~60 MPa for 10 min. For Mg_1−x_Cu_x_Ag_0.97_Sb_0.99_, we used the two-step ball-milling method and then did SPS at 573 K^22,35^.

### Phase and microstructure characterizations

The phase structure was characterized by powder X-ray diffraction (XRD, SmartLab3, Rigaku) with Cu K_α_ radiation. DSC measurement (Netzsch STA 449F1 Jupiter) was performed in an N_2_ atmosphere at a heating rate of 15 K/min. The microstructure and composition analysis were characterized using a field emission scanning electron microscope (FESEM, Hitachi S-4800) equipped with an energy dispersive spectrometer (EDS, Horiba EMAXEvolution X-Max).

### Material property characterizations

Bar samples with a dimension of around 2.5 mm × 2.5 mm × 9 mm were cut from the pressed disks and used for simultaneous measurement of electrical resistivity (*ρ*) and Seebeck coefficient (*S*) on a commercial system (ULVAC ZEM-3). The thermal conductivity *κ*_*tot*_ was calculated using *κ*_*tot*_ = *DC*_*p*_*d*, where *D*, *C*_*p*_, and *d* are the thermal diffusivity, specific heat capacity, and density, respectively. The thermal diffusivity coefficient (*D*) and the specific heat capacity (*C*_*p*_) were concurrently measured for the disk sample on a laser flash system (Netzsch LFA 467, Germany) with a pyroceram disk as a reference sample. For example, *C*_*p*_ measurement result of Mg_3.2_Bi_1.5_Sb_0.498_Te_0.002_Cu_0.01_ samples sintered at 973 K is in good consistency with the corrected Dulong-Petit law in this material system^60^ (Fig. S15). The sample density (*d*) was determined by the Archimedes method. The room-temperature Hall coefficient *R*_*H*_ was measured using the PPMS (Physical Properties Measurement System, Quantum Design) with the AC transport option. The data were obtained with a magnetic field sweeping from −5 T to +5 T. The Hall carrier concentration (*n*_*H*_) was obtained by *n*_*H*_ = 1/*eR*_*H*_ and the Hall carrier mobility (*μ*_H_) was calculated by *σ* = *eμ*_*H*_*n*_*H*_, where *e* is the electronic charge and *σ* the electrical conductivity. The abovementioned measurement results are directly obtained from one sintering disk.

### Module fabrication, performance measurement, and stimulation method

Thermoelectric legs of n-type Mg_3.2_Bi_1.5_Sb_0.498_Te_0.002_Cu_0.01_ sintered at 973 K and p-type Mg_0.99_Cu_0.01_Ag_0.97_Sb_0.99_ legs with interfacial layer were fabricated by the one-step SPS using the same condition as previously mentioned. Fe powder and Ag powder for n-type and p-type contact layer was used as the starting material, respectively. These obtained sandwich disks were grinding, polishing, and dicing. The cross-section of legs is 2.0 mm × 2.0 mm with a total length of 3 mm. The lengths of the thermoelectric elements and interfacial layer are 1.75 mm and 0.8 mm, respectively.

The power-generation performance measurement is similar to previous reports^33,61^. The eight-couple p- and n-type legs were alternately positioned onto the Cu substrate (18 mm × 15 mm × 1 mm), where thick Cu patterns were printed onto the heat-conducting polymer film. The legs were interconnected by Cu electrodes, in which InGa eutectic alloy was used for soldering. The power-generation performance was measured in a vacuum on a home-built apparatus with a hot-side temperature of 293, 313, 333, 353, and 373 K, in which the cold-side temperature was fixed at 278 K. The cold-side temperatures *T*_*c*_ and hot-side temperature *T*_*h*_ were measured by using thermocouples embedded in the ceramic plates, respectively. A Cu block with known thermal conductivity (*κ*_*Cu*_) and a fixed cross-sectional area *A*_*Cu*_ was used to measure the heat flow. The heat flow out from the cold-side (*Q*_*out*_) was calculated based on Fourier’s law:

2 $${Q}_{out}\,=\,{\kappa }_{Cu}\bigtriangleup T({A}_{Cu}/{L}_{Pt})$$

where *L*_*Pt*_ is the vertical distance of Pt resistance thermometer in the Cu block and *ΔT* is the temperature difference along the direction of heat flow. The conversion efficiency *η* was calculated using the following equation:

3 $$\eta \,=\,\frac{P}{P\,+\,{Q}_{out}}$$

where *P* is the electrical power output measured by a direct current electronic load.

The cooling-performance fabricated modules were measured keeping a hot-side temperature to be 373 K in a vacuum (10^−2^–10^−3^ Pa) on a home-built testing system. The setup is similar with that for power-generation measurement except that hot- and cold-side is upside down and that the Cu block for evaluating heat flow is located above the module.

The three-dimensional finite-element simulations of power-generation and cooling performance was performed with COMSOL Multiphysics^®^ software in the Heat Transfer Module. A geometrical model was built in the software interface to represent module with an identical geometry and dimension. The module structure from top to bottom is AlN ceramic 1 mm, graphite foil 0.1 mm, InGa layer 0.03 mm, thermoelectric element 3.35 mm, InGa layer 0.03 mm, Cu foil 0.21 mm, insulation foil 0.08 mm, grease 0.03 mm, and AlN ceramic 1 mm. Herein, the electrical and thermal contact resistances between these interfaces, as well as resistances of Cu electrodes are not considered in our simulation model. The measured temperature-dependent thermoelectric properties of n-type Mg_3.2_Bi_1.5_Sb_0.498_Te_0.002_Cu_0.01_ and p-type Mg_0.99_Cu_0.01_Ag_0.97_Sb_0.99_ were used for simulation, while those for the Ag and Fe contact layers were assumed to be constant.

### Reporting summary

Further information on research design is available in the Nature Research Reporting Summary linked to this article.

## Supplementary information

Supplementary Information

Reporting Summary

Lasing Reporting Summary

## Data Availability

The data that support the findings of this study are available from the corresponding author upon reasonable request.
