# Supplementary material for: Maximizing the performance of n-type Mg3Bi2 based materials for room-temperature power generation and thermoelectric cooling
Source: Nat Commun. 2022 Mar 2;13:1120. doi: 10.1038/s41467-022-28798-4 (PMC8891317; doi:10.1038/s41467-022-28798-4)
Supplement: Supplementary file 1 — Supplementary Information [file 41467_2022_28798_MOESM1_ESM.pdf]

Supporting Information for

Maximizing the Performance of n-Type Mg_3Bi_2 Based Materials for Room-Temperature Power Generation and Thermoelectric Cooling

Zihang Liu[a], Weihong Gao[a], Hironori Oshima[b], Kazuo Nagase[b], Chul-Ho Lee[b], and Takao Mori[a,c*]

[a] WPI Center for Materials Nanoarchitectonics (WPI-MANA), National Institute for Materials Science (NIMS), Namiki 1-1, Tsukuba 305-0044, Japan

[b] National Institute of Advanced Industrial Science and Technology (AIST), Tsukuba, Ibaraki, 305-8568, Japan

[c] Graduate School of Pure and Applied Sciences, University of Tsukuba, Tennodai 1-1-1, Tsukuba 305-8671, Japan

These authors contributed equally: Zihang Liu and Weihong Gao. To whom correspondence should be addressed. E-mail: MORI.Takao@nims.go.jp

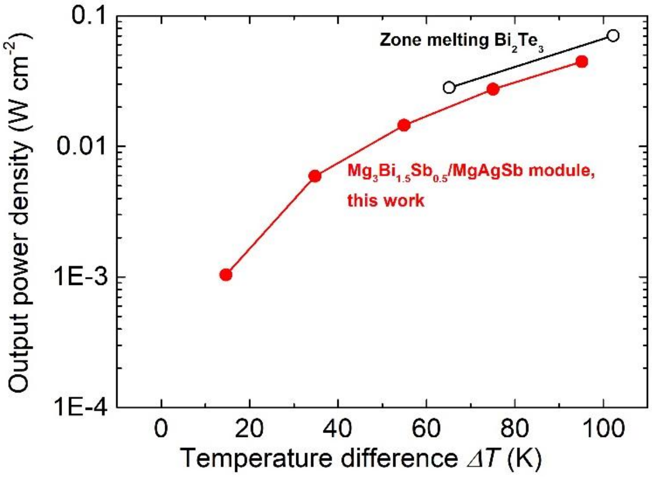

Figure S1. Comparison of the calculated output power density between our $Mg_3Bi_{1.5}Sb_{0.5}$/MgAgSb module and zone-melting Bi_2Te_3 module as a function of temperature difference ΔT

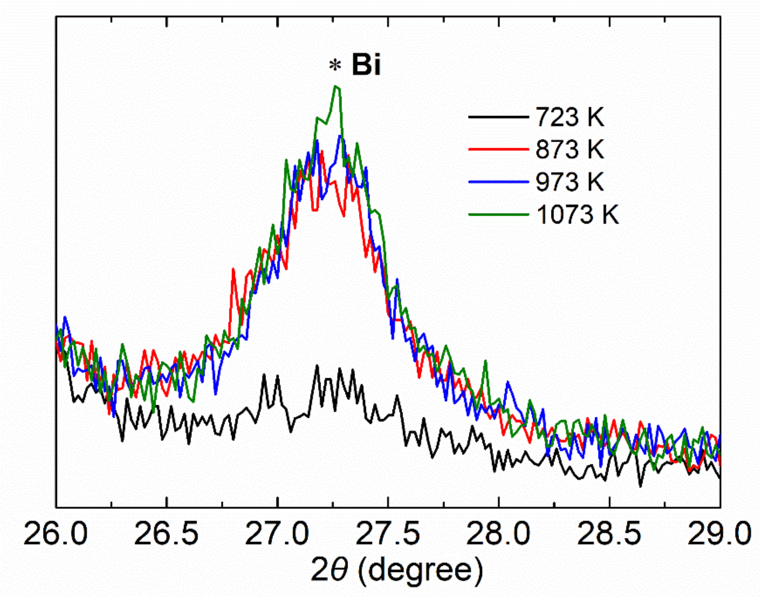

Figure S2. The enlarged XRD pattern of $Mg_{3.2}Bi_{1.5}Sb_{0.498}Te_{0.002}Cu_{0.01}$ samples at different sintering temperatures (723 K, 873 K, 973 K, and 1073 K).

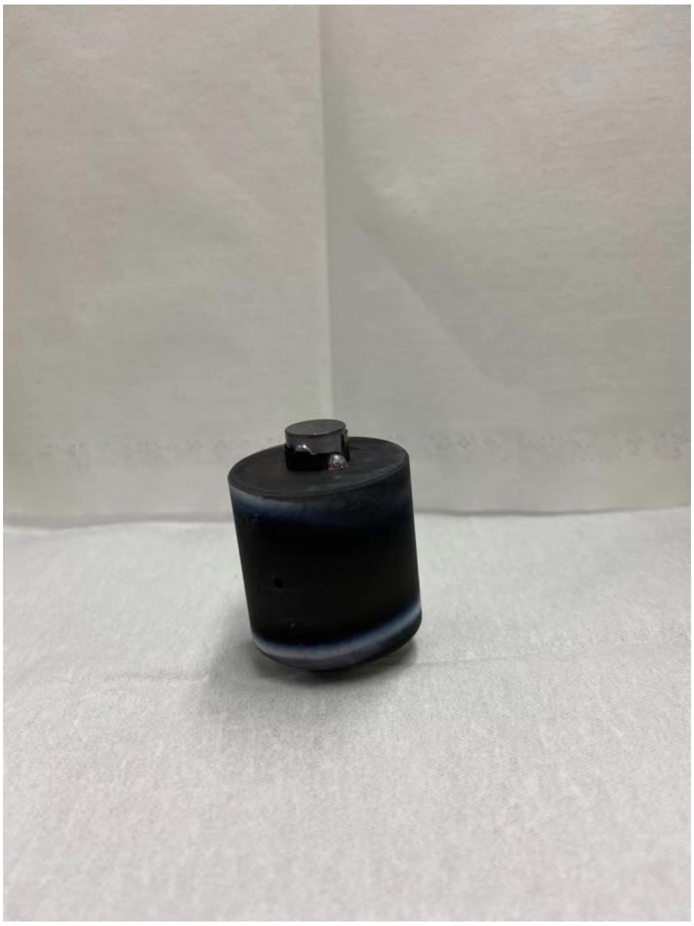

Figure S3. The image of the evaporation phenomenon and the melting phase during the sintering process at 1073 K.

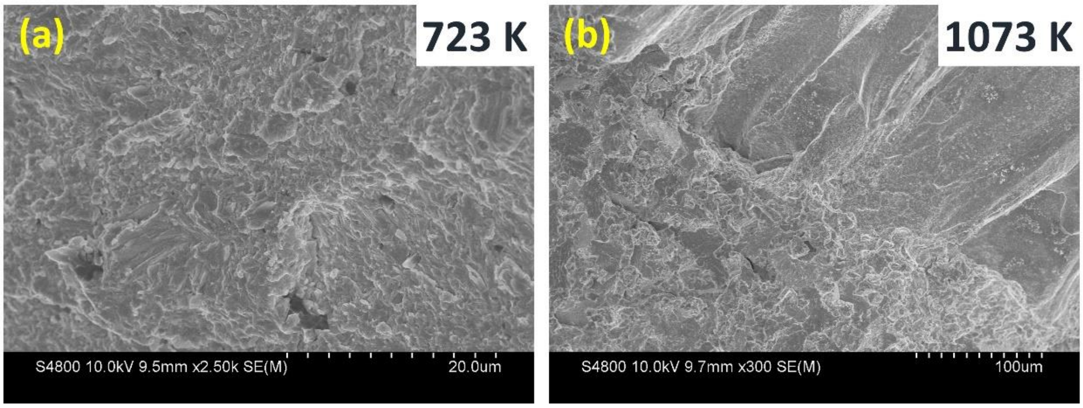

Figure S4. (a) and (b) The grain morphology at 723 K and 1073 K, respectively.

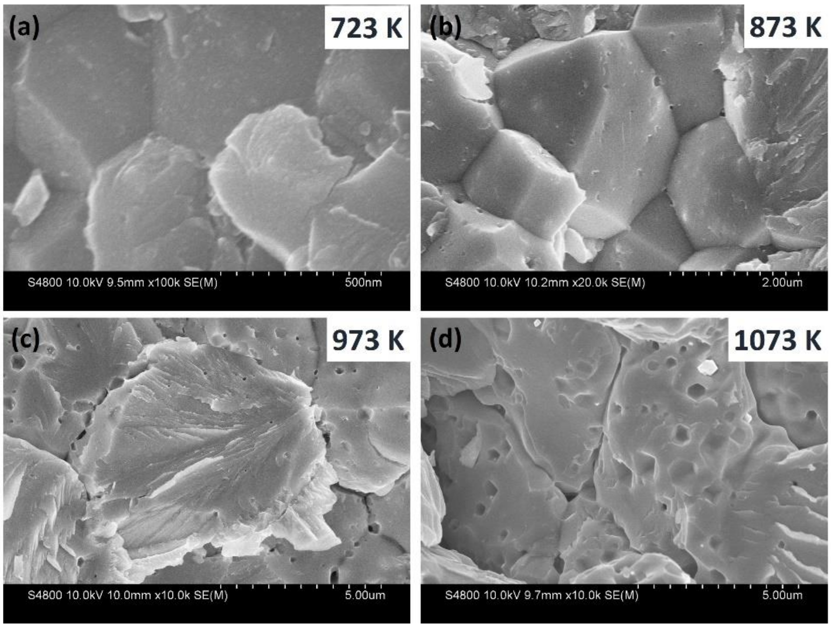

Figure S5. SEM images about these pores inside $Mg_{3.2}Bi_{1.5}Sb_{0.498}Te_{0.002}Cu_{0.01}$ samples at different sintering temperatures. (a) 723 K, (b) 873 K, (c) 973 K, and (d) 1073 K.

Table S1. Sample density of $Mg_{3.2}Bi_{1.5}Sb_{0.498}Te_{0.002}Cu_{0.01}$ samples at different sintering temperatures (723 K, 873 K, 973 K, and 1073 K)

Sample label	sample density (g cm^{-3})
SPS-723 K	5.29
SPS-873 K	5.40
SPS-973 K	5.36
SPS-1073 K	5.08

Table S2. The measured atomic composition ration of $Mg_{3.2}Bi_{1.5}Sb_{0.498}Te_{0.002}Cu_{0.01}$ samples at different sintering temperatures by SEM+EDS

Sample label	Mg	Bi	Sb
723 K	60.74	29.95	9.31
873 K	60.19	30.31	9.50
973 K	59.43	31.03	9.54
1073 K	58.41	31.76	9.83

Table S3. Drift mobility μ_D and the density of states effective mass m^* of $Mg_{3.2}Bi_{1.5}Sb_{0.498}Te_{0.002}Cu_{0.01}$ samples at different sintering temperatures (723 K, 873 K, 973 K, and 1073 K)

Sample label	drift mobility μ_D ($cm^2\ V^{-1}\ s^{-1}$)	density of states effective mass m^* (m_0)
SPS-723 K	33.36	1.87
SPS-873 K	175.15	0.92
SPS-973 K	316.85	0.88
SPS-1073 K	19.20	0.51

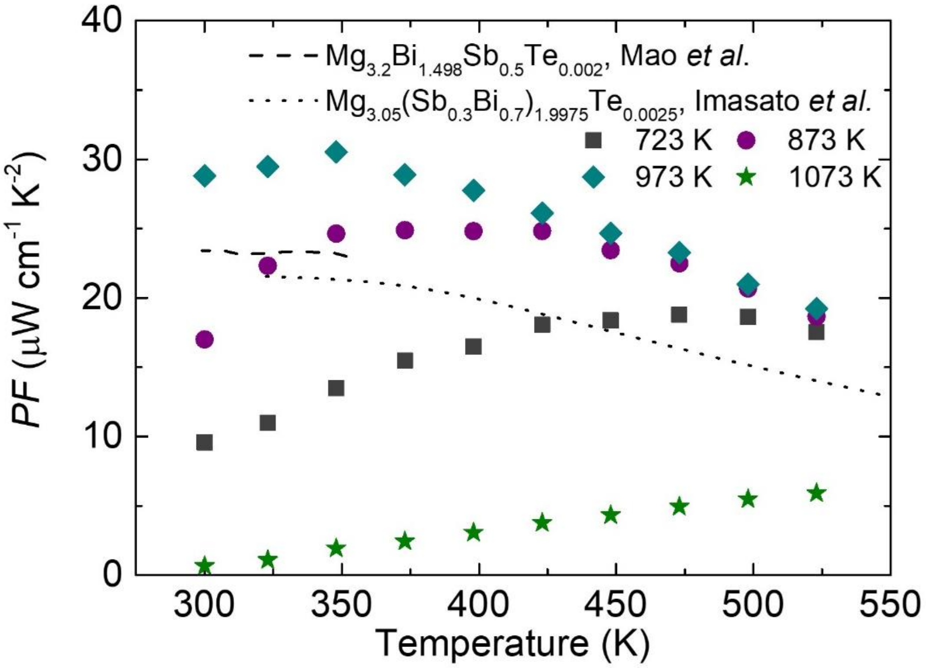

Figure S6. Temperature-dependent power factor of $Mg_{3.2}Bi_{1.5}Sb_{0.498}Te_{0.002}Cu_{0.01}$ samples at different sintering temperatures (723 K, 873 K, 973 K, and 1073 K),), in comparison to the data from Mao *et al*. and Imasato *et al*.[1,2]

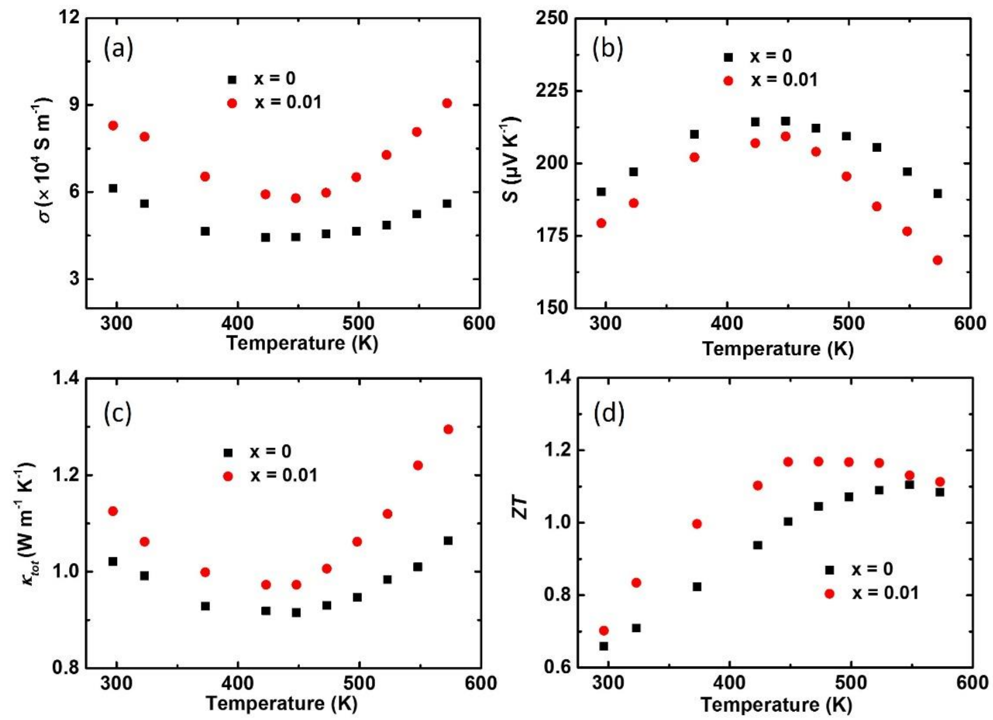

Figure S7. Thermoelectric properties of $Mg_{1-x}Cu_xAg_{0.97}Sb_{0.99}$ (x = 0 and 0.01). (a), (b), (c), and (d) Temperature-dependent electrical conductivity σ, Seebeck coefficient S, total thermal conductivity κ_{tot}, and zT, respectively[3]. (Submitted)

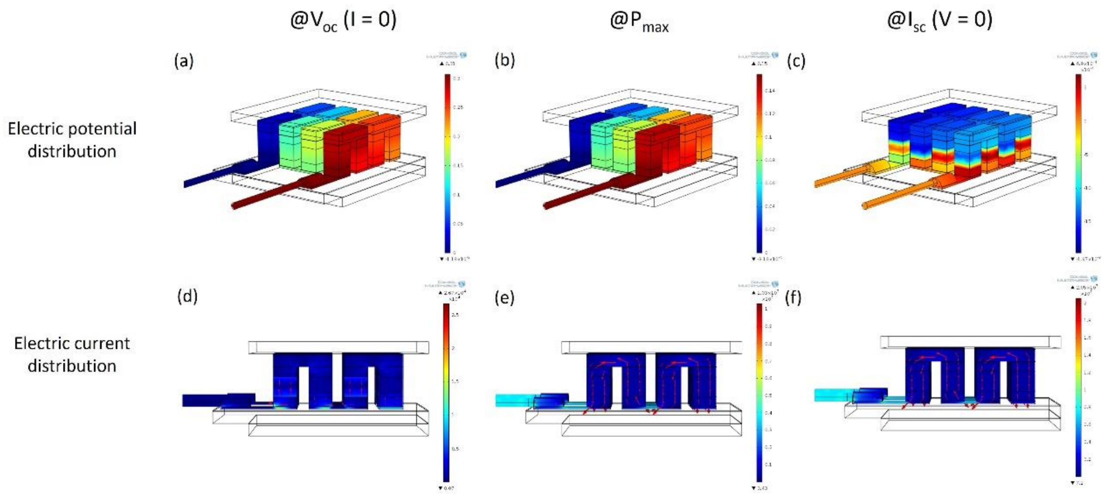

Figure S8. Three-dimensional finite-element simulations of power-generation performance with COMSOL Multiphysics® software. (a), (b) and (c) Electrical potential distribution when measuring the open-circuit voltage V_{oc}, maximum power output P_{max}, and short-circuit current I_{sc}, respectively. (d), (e) and (f) Electric current distribution when measuring the open-circuit voltage V_{oc}, maximum power output P_{max}, and short-circuit current I_{sc}, respectively.

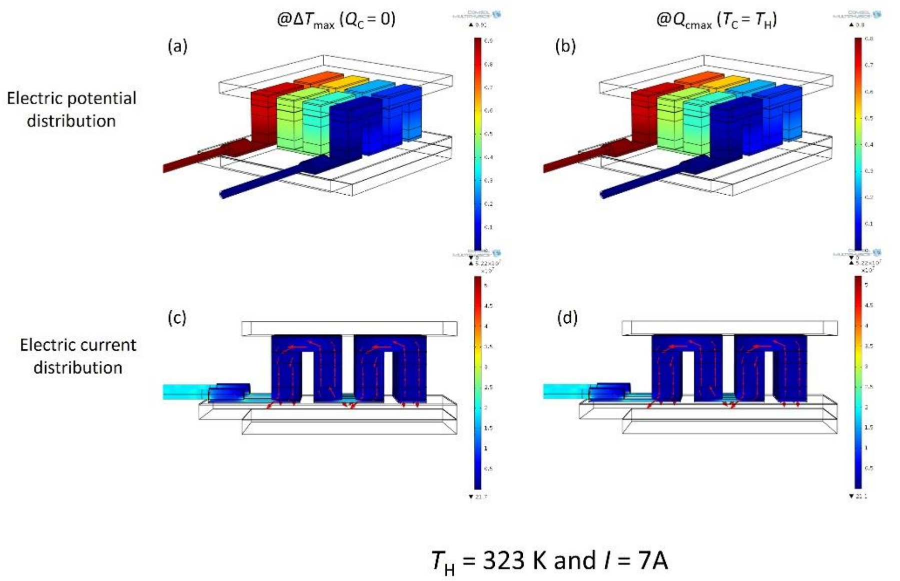

Figure S9-1. Three-dimensional finite-element simulations of cooling performance with COMSOL Multiphysics® software at the hot side T_h of 323 K and the working current 7 A. (a) and (b) Electrical potential distribution when measuring the maximum temperature difference ΔT_{max} and the maximum cooling power Q_{cmax}, respectively. (c) and (d) Electric current distribution w when measuring the maximum temperature difference ΔT_{max} and the maximum cooling power P_{max}, respectively.

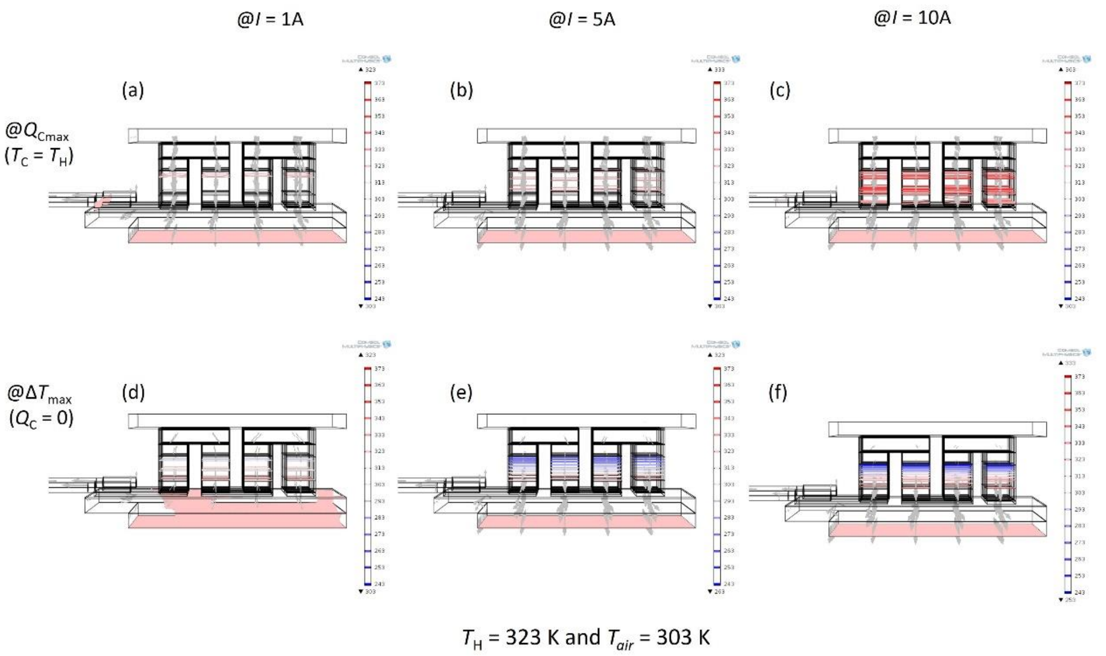

Figure S9-2. Three-dimensional finite-element simulations of cooling performance with COMSOL Multiphysics® software at the hot side T_h of 323 K and the air

temperature T_{air} of 303 K. (a), (b), and (c) Heat flow distribution when measuring the maximum cooling power Q_{cmax} at the working current I of 1 A, 5 A, and 10 A, respectively. (d), (e), and (f) Heat flow distribution when measuring the maximum temperature difference ΔT_{max} at the working current I of 1 A, 5 A, and 10 A, respectively.

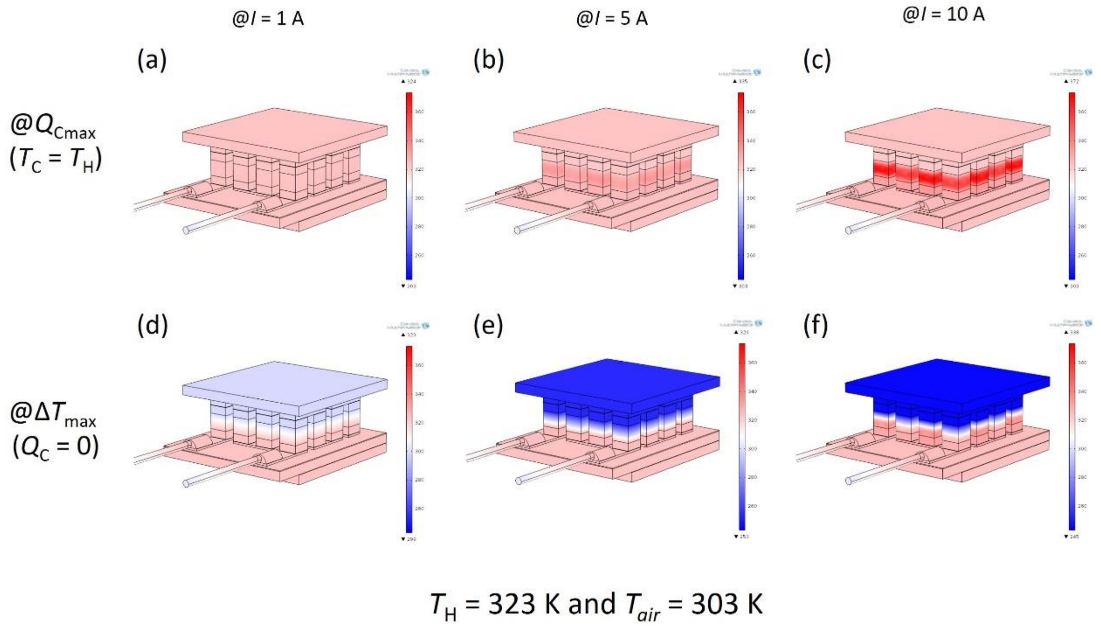

Figure S9-3. Three-dimensional finite-element simulations of cooling performance with COMSOL Multiphysics® software at the hot side T_h of 323 K and the air temperature T_{air} of 303 K. (a), (b), and (c) Temperature distribution when measuring the maximum cooling power Q_{cmax} at the working current I of 1 A, 5 A, and 10 A, respectively. (d), (e), and (f) Temperature distribution when measuring the maximum temperature difference ΔT_{max} at the working current I of 1 A, 5 A, and 10 A, respectively.

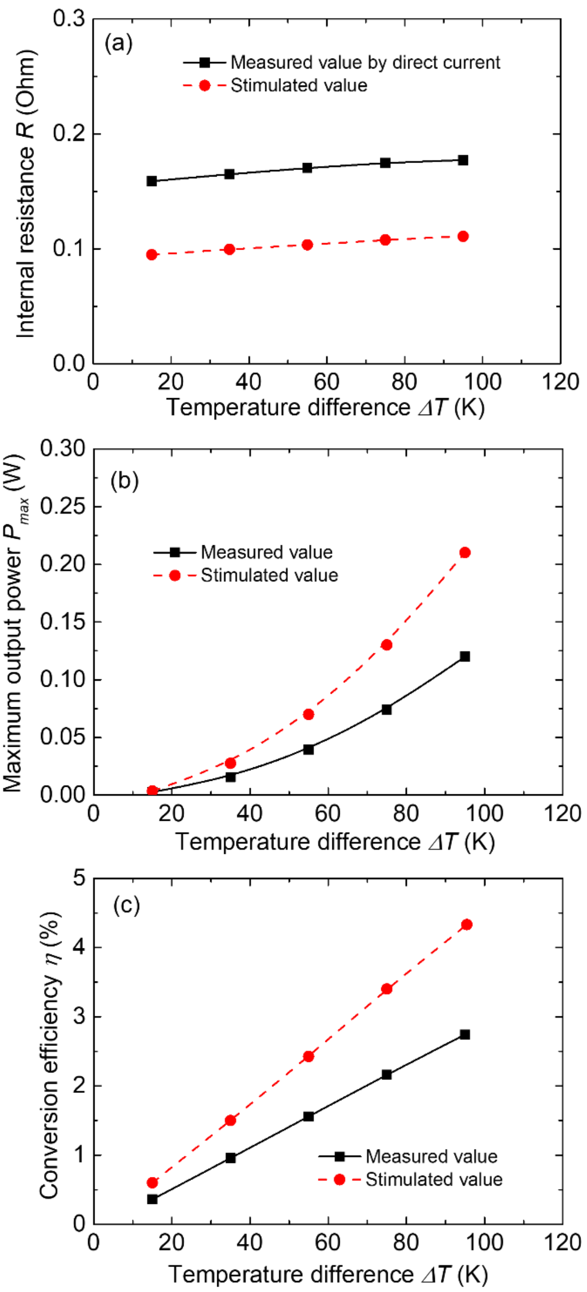

Figure S10. Comparison of the measured value and stimulated value of our fabricated module for power generation using COMSOL Multiphysics ® software. (a) The internal resistance R, (b) the maximum output power P_{max}, and (c) the conversion efficiency η as a function of the temperature difference ΔT.

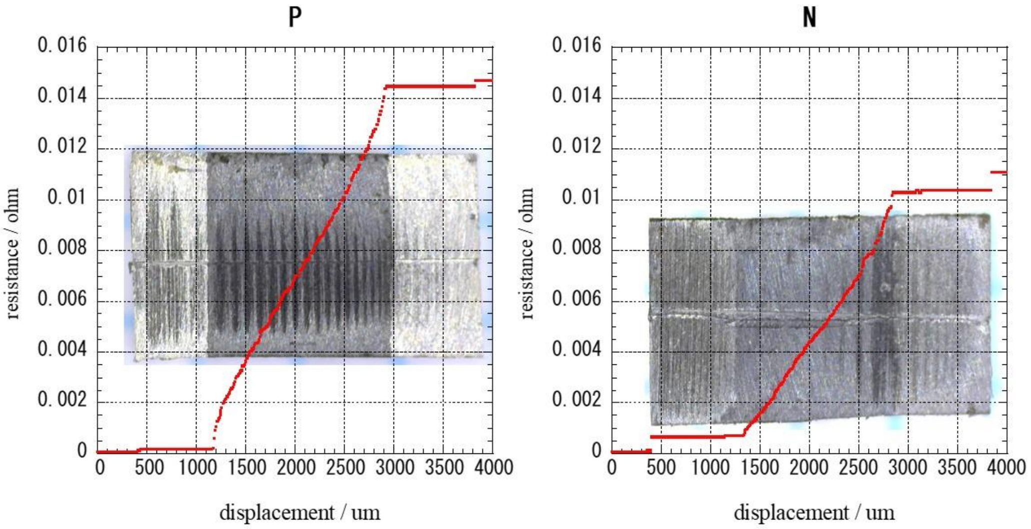

Figure S11. (a) and (b) The measured contact resistance of the sandwich structure Ag/MgAgSb/Ag and Fe/$Mg_{3.2}Bi_{1.5}Sb_{0.5}$/Fe interface, respectively.

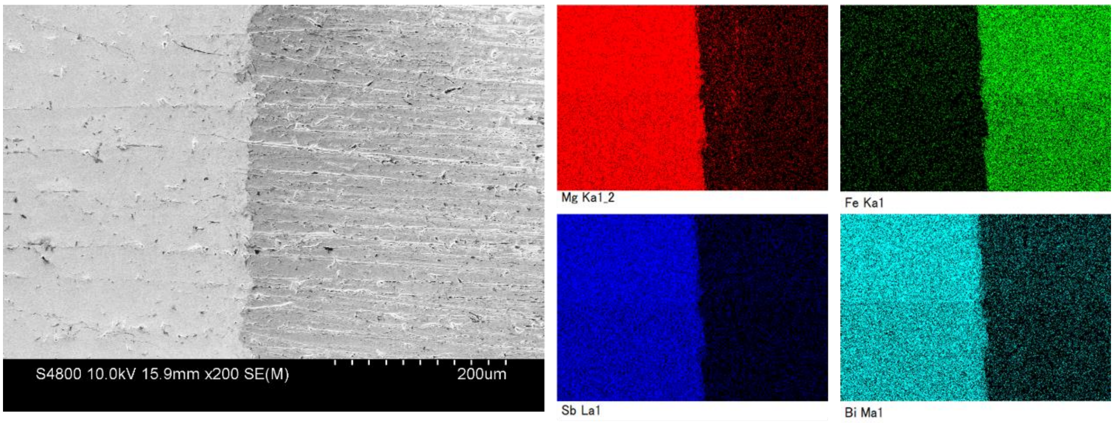

Figure S12. The surface morphology and the corresponding EDS mapping around the interface between the interfacial layer and element material for n-type $Mg_{3.2}Bi_{1.5}Sb_{0.498}Te_{0.002}Cu_{0.01}$.

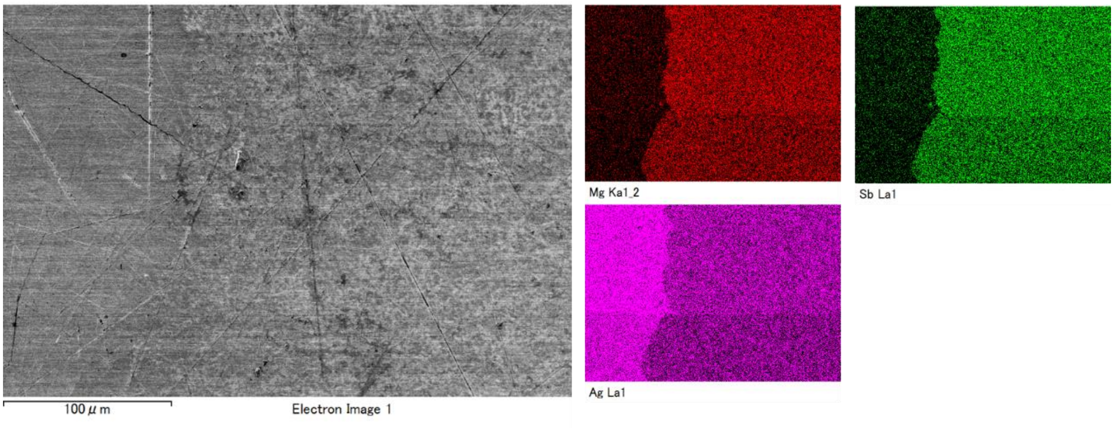

Figure S13. The surface morphology and the corresponding EDS mapping around the interface between the interfacial layer and element material for p-type α-$Mg_{0.99}Cu_{0.01}Ag_{0.97}Sb_{0.99}$.

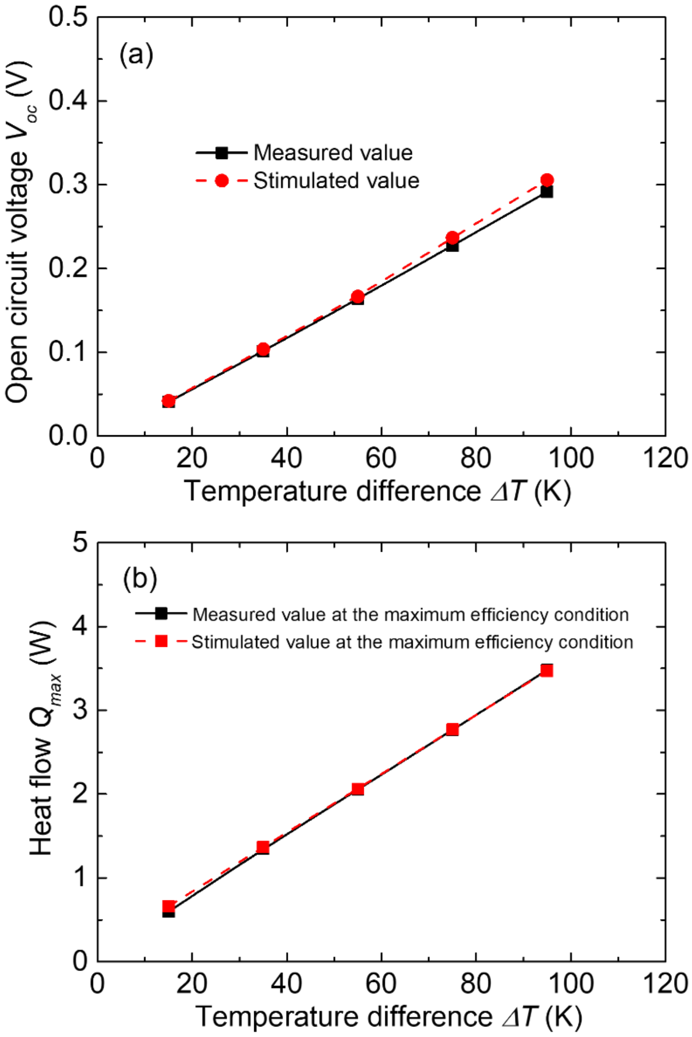

Figure S14. Comparison of the measured value and stimulated value of our fabricated module for power generation using COMSOL Multiphysics ® software. (a) The open-circuit voltage V_{oc} and (b) the maximum heat dissipated from the cold side of the module Q_{max} as a function of the temperature difference ΔT.

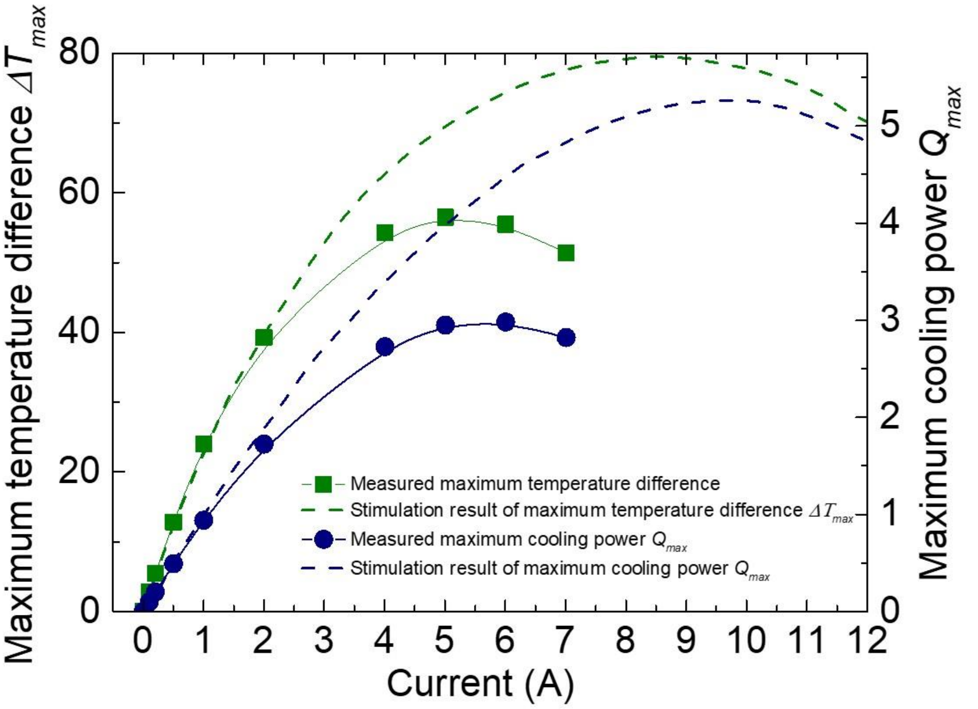

Figure S15. Comparison of the measured value and stimulated value in our fabricated module for thermoelectric cooling using COMSOL Multiphysics® software, including the maximum temperature difference ΔT_{max} and the maximum cooling power Q_{max} as a function of electric current I at the hot-side temperature T_h of 323 K.

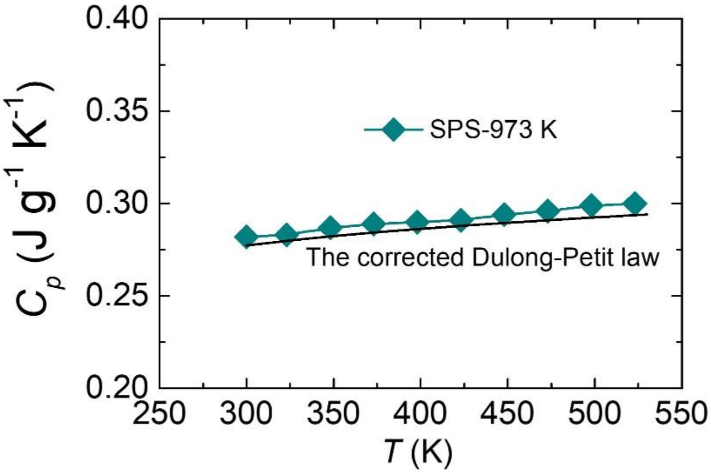

Figure S16. Temperature-dependent heat capacity C_p of $Mg_{3.2}Bi_{1.5}Sb_{0.498}Te_{0.002}Cu_{0.01}$ sample (SPS 973 K), in comparison to the corrected Dulong-Petit law from Agne *et al.*[4]

Reference

1 Mao, J. *et al.* High thermoelectric cooling performance of n-type Mg3Bi2-based materials. *Science* **365**, 495-498 (2019).

2 Imasato, K., Kang, S. D. & Snyder, G. J. Exceptional thermoelectric performance in Mg3Sb0.6Bi1.4 for low-grade waste heat recovery. *Energy Environ. Sci.* **12**, 965-971 (2019).

3 Liu, Z. H. *et al.* Demonstration of ultrahigh thermoelectric efficiency of ~7.3% in Mg3Sb2/MgAgSb module for low-temperature energy harvesting. *Joule* **5**, 1196-1208 (2021).

4 Agne, M. T. *et al.* Heat capacity of Mg3Sb2, Mg3Bi2, and their alloys at high temperature. *Mater. Today. Phys.* **6**, 83-88 (2018).
